# Injury mortality in South Africa: a 2009 and 2017 comparison to track progress to meeting sustainable development goal targets

**DOI:** 10.1080/16549716.2024.2377828

**Published:** 2024-08-15

**Authors:** Megan Prinsloo, Shibe Mhlongo, Rifqah A. Roomaney, Lea Marineau, Thakadu A. Mamashela, Bianca Dekel, Debbie Bradshaw, Lorna J. Martin, Carl Lombard, Rachel Jewkes, Naeemah Abrahams, Richard Matzopoulos

**Affiliations:** a Burden of Disease Research Unit, South African Medical Research Council, Cape Town, South Africa; b Division of Public Health Medicine, School of Public Health, Faculty of Health Sciences, University of Cape Town, Cape Town, South Africa; c Institute for Lifecourse Development, Faculty of Education, Health & Human Sciences, University of Greenwich, Park Row, UK; d Gender and Health Research Unit, South African Medical Research Council, Cape Town, South Africa; e School of Nursing, Johns Hopkins University, Baltimore, USA; f Department of Forensic Medicine, Faculty of Health Sciences, University of Limpopo, Limpopo, South Africa; g Division of Forensic Medicine, Faculty of Health Sciences, University of Cape Town, Cape Town, South Africa; h Biostatistics Unit, South African Medical Research Council, Cape Town, South Africa; i Division of Epidemiology and Biostatistics, Department of Global Health, Stellenbosch University, Cape Town, South Africa; j Office of the Executive Scientist, South African Medical Research Council, Cape Town, South Africa; k Division of Social and Behavioural Sciences, School of Public Health, Faculty of Health Sciences, University of Cape Town, Cape Town, South Africa

**Keywords:** Injuries, violence, homicide, road traffic, suicide, unintentional, Sustainable Development Goals

## Abstract

**Background:**

Injuries, often preventable, prompted urgent action within the United Nations’ 2030 Agenda for Sustainable Development Goals (SDGs) to improve global health. South Africa (SA) has high rates of injury mortality, but accurate reporting of official national data is hindered by death misclassification.

**Objective:**

Two nationally representative surveys for 2009 and 2017 are utilised to assess SA’s progress towards SDG targets for violence and road traffic injuries, alongside changes in suicide and under-5 mortality rates for childhood injuries, and compare these estimates with those of the Global Burden of Disease for SA.

**Methods:**

The surveys utilised multi-stage, stratified cluster sampling from eight provinces, with mortuaries as primary sampling units. Post-mortem files for non-natural deaths were reviewed, with additional data from the Western Cape. Age-standardised rates, 95% confidence intervals (CIs), and incidence rate ratios (IRRs) were calculated for manner of death rate comparisons and for age groups.

**Results:**

The all-injury age-standardised mortality rate decreased significantly between 2009 and 2017. Homicide and transport remained the leading causes of injury deaths, with a significant 31% decrease in road traffic mortality (IRR = 0.69), from 36.1 to 25.0 per 100 000 population.

**Conclusions:**

Despite a reduction in SA’s road traffic mortality rate, challenges to achieve targets related to young and novice drivers and male homicide persist. Achieving SA’s injury mortality SDG targets requires comprehensive evaluations of programmes addressing road safety, violence reduction, and mental well-being. In the absence of reliable routine data, survey data allow to accurately assess the country’s SDG progress through commitment to evidence-based policymaking.

## Paper context

**Main findings:**

The significant decrease in South Africa’s injury mortality rates between 2009 and 2017 appears to largely be driven by the significant 31% decrease in road traffic mortality rates.

**Added knowledge:**

The 2009 and 2017 survey comparison provides an enhanced understanding of the profile for injury-related deaths, compared to misclassified vital statistics data, to track progress towards reaching Sustainable Development Goals.

**Global health impact for policy and action:**

The significant reduction in road traffic mortality across all age groups suggests South Africa is making progress towards Sustainable Development Goal Target 3.6 for road safety. However, reducing violence, suicide, and newborn and under-5 injury mortality requires more targeted interventions.

## Background

Injuries are predictable and hence preventable, as shown through evidence-based intervention guidelines and prevention strategies [1–5]. The large global magnitude of injuries and violence formed the basis for their inclusion in an urgent call for action by the United Nations (UN) in 2015, as part of a 2030 Agenda for Sustainable Development. The Sustainable Development Goal (SDG) Target 3.6 initially aimed to halve the number of global road traffic deaths and injuries by 2020 [6], but a progress report from the UN Statistics Division showed that this was not met [7]. In addition, SDG Target 16.1 aims to significantly reduce all forms of violence and related death rates everywhere [8]. The indicators for the respective goals include the number and rate of road traffic deaths and homicide as a measure of progress for each UN member country. The suicide mortality rate, as an indicator for SDG Target 3.4, aims to reduce premature mortality from non-communicable diseases, through promoting mental health and well-being. Childhood deaths from injuries are monitored as an indicator for SDG Target 3.2, which aims to end preventable deaths of newborns and children under 5 years of age. National research is needed in South Africa (SA), with its competing health priorities [9,10], to establish progress towards meeting the targets and showing this in the early years of the SDGs is the focus of this paper.

South Africa’s high injury mortality rate has declined, largely due to a significant decrease in homicide rates between 2000 and 2010. However, high injury mortality rates remain, especially for road traffic-related deaths and homicides, of which the latter have been rising in recent years [11,12]. Official national civil registration and vital statistics data for injuries are not accurately reported, largely due to misclassification of deaths [13]. This poses a challenge to any monitoring of injury mortality trends and progress to reaching the SDG’s, not only in SA but in other low-to-middle-income countries (LMICs), who carry 90% of the global injury mortality burden [14]. The World Health Organization (WHO) developed a manual for implementing mortuary-based injury mortality surveillance in LMICs [15]. However, a process evaluation in five LMICs [16] revealed that successful implementation requires training forensic pathologists and data capturers. In addition, demonstrating the data’s usefulness to secure government funding is needed. Resource-constrained countries are often reliant on modelled estimates from Global Burden of Disease (GBD) studies, to monitor progress to reaching the SDGs [17]. This is not the ideal, as shown in a comparative analysis of our nationally representative survey of injury-related deaths for 2009, collected from mortuaries, with that of GBD estimates modelled from SA vital statistics data. The comparative analysis for 2009 identified an underestimation for homicide, road traffic, and suicide rates for SA [12]. This demonstrates the importance of conducting empirical mortuary-based studies to monitor progress towards meeting the SDGs.

Since the 2009 survey, a second study for 2017 further retrieved epidemiological information on injury deaths from mortuary data, which enabled the monitoring of changes in injury mortality rates, and a more specifically detailed discussion in publications on changes in male homicide [18], femicide [19], child homicide [20–22], and victim–perpetrator relationships for male homicide [23]. This paper includes data of the aforementioned citations but will not be discussed in detail. In this study, we present analysis of the 2009 and 2017 surveys, with more specific detail on the mechanism of injury-related deaths, than misclassified vital statistics data [13,24]. We aim to firstly indicate progress towards achieving the two SDG targets for violence and road traffic injuries as the leading contributors to injury deaths for South Africa [24], as well as to highlight changes to the suicide rate and under-5 mortality rate for childhood injuries. Secondly, we compare our survey estimates, to that of GBD estimates for South Africa.

## Method

### Study design and sampling

The 2009 and 2017 surveys were retrospective descriptive studies that used two strategies to collect nationally representative data on non-natural deaths. A nationally representative sample of mortuaries was chosen from eight provinces. The data for the ninth province, Western Cape (WC), was obtained from the health department, which maintains a surveillance system with compatible data for all mortuaries in the province.

For 2009, sampling was stratified by metro- and non-metro areas, and a sampling frame of 106 mortuaries (57 274 postmortem report files) was used to draw a sample of 45 mortuaries with an expected sample of 22 733 records for the database. The 2009 survey has since been published [12] and will be included in the analyses for this paper. The recently completed 2017 survey was stratified by province. A sampling frame of 121 mortuaries (58 641 files) was used to draw a sample of 65 mortuaries, with an expected sample of 26 161 records. For both surveys, the mortuaries were stratified by size based on the sampling frame from the Female and Child Homicide study [19]. For the small- and medium-sized mortuaries (≤500 bodies and 501–1 500 bodies respectively), all files were included. For large mortuaries (>1 500 bodies), every second file was selected; however, for homicide cases, all files for child and adult females were included.

### Data collection

Registers and post-mortem files were reviewed for patients who died of a non-natural death and required a post-mortem examination at a medico-legal mortuary [24]. Data were collected for deaths from 1 January 2017 to 31 December 2017 using a Tablet for data capture on a Kobotools platform [25]. Information on province, mortuary, demographics, date of death, and apparent manner of death (homicide, suicide, transport, other unintentional and undetermined) were captured. Mechanisms of injury (i.e. firearm, sharp force, hanging, poisoning, driver, burns, etc.) were captured in accordance with the International Statistical Classification of Diseases (ICD) version 10 [26]. Fieldwork occurred between 20 January-25 March 2020, placed on hold as a result of COVID-19 lockdown restrictions and completed between 30 June-3 July 2020.

### Data cleaning and analysis

Data were cleaned and analysed using Stata version 17 (Stata Corporation, College Station, Texas, USA). The analysed data considered the survey design and the sampling weights of mortuaries, assigned within survey strata using province and mortuary size.

We investigated a large number of deaths from undetermined manner for 2017, to identify and exclude probable natural deaths. This process included the merging of the Rapid Mortality Surveillance (RMS) database [27] with the 2017 injury mortality database. The RMS database collates information on cause and distinguishes by natural and non-natural deaths in South Africa from the National Population Register, held by the Department of Home Affairs [27]. Hence, the undetermined deaths within the 2017 survey data, which could be linked to natural deaths in the RMS as an alternative source, were excluded. Natural deaths were further excluded from the 2017 undetermined deaths, by screening the injury mechanisms and the comments provided in free text fields.

Age-standardised rates and 95% confidence intervals (CIs) for each manner of death and specific injury mechanisms were calculated using 2009 and 2017 population estimates [28]. We used the WHO world standard population weights [29] and missing age was redistributed. Age-specific rates were calculated for manner of death by age groups. Incidence rate ratios (IRRs) and 95% CIs were calculated to compare the two survey estimates. The standard errors for the IRRs considered the design effect of each survey.

For the comparison with the 2017 GBD estimates for South Africa [30], our survey’s undetermined deaths for 2017 were proportionally redistributed across manner of death categories. The age-standardised rates were adjusted, by multiplying a scaling factor to the rate for each manner of death. The scaling factor was calculated in Microsoft Excel [31], using the following equation:

$$\mathrm{Scaling}\;\;\mathrm{factor}=\;\frac{\mathrm{Age}\;\;\mathrm{standardised}\;\;\mathrm{death}\;\;\mathrm{rate}\;\;\mathrm{for}\;\;\mathrm{all}\;\;\mathrm{injuries}}{\mathrm{Age}\;\;\mathrm{standardised}\;\;\mathrm{death}\;\;\mathrm{rate}\;\;\mathrm{for}\;\;\mathrm{all}\;\;\mathrm{injuries}\;\;-\;\;\mathrm{Undeterminded}\;\;\mathrm{age}\;\;\mathrm{standardised}\;\;\mathrm{death}\;\;\mathrm{rate}}$$

### Ethics

The South African Medical Research Council’s Human Research Ethics Committee provided ethical approval for this study (HREC EC008–5/2018).

## Results

For 2009, 24 197 non-natural deaths were included (i.e. 18 241 non-natural deaths recorded from fieldwork across 8 provinces and 5 956 non-natural deaths from the WC). These reflected a weighted total of 52 493 non-natural deaths once sampling weights were applied. For 2017, a total of 30 996 non-natural deaths were included (i.e. 22 822 non-natural deaths from fieldwork in 8 provinces and 8 174 deaths from the WC), and a total of 29 944 death records were weighted, to reflect an estimate of 53 288 non-natural deaths for analysis.

There was a decrease in the age-standardised mortality rate for all injuries between 2009 and 2017, from 109 per 100 000 population (95% CI: 97.1–121.0) to 95 per 100 000 (95% CI: 89.7–100.9), for the weighted estimate of 53 288 deaths (95% CI: 49 964–56 613) for 2017 (Table 1). The calculated IRR estimate showed that this decrease was significant (IRR = 0.87, 95% CI: 0.86–0.88).

**Table 1.** Homicide, transport, suicide, and other unintentional injury mortality rates for 2009 and 2017 by injury mechanism (weighted).

Apparent manner of death and injury mechanism	2009*		2017		
	Persons^a^ (*N* = 52 493)		Persons^a^ (*N* = 53 288)		
	Total No. (95% CI)	Mortality rate per 100 000 population (95% CI)	Total No. (95% CI)	Mortality rate per 100 000 population (95% CI)	Incidence rate ratio: 2017/2009: (95% CI)
**All Injuries**	**52 493 (46 930–58 057)**	**109.0 (97.1–121.0)**	**53 288 (49 964–56 613)**	**95.3 (89.7–100.9)**	**0.87 (0.86–0.88)**
**Homicide**	**19 028 (16 852–21 204)**	**38.4 (33.8–43.0)**	**19 477 (18 153–20 800)**	**34.0 (31.6–36.4)**	**0.89 (0.87–0.92)**
- Firearm discharge	5 513 (4 937–6 090)	11.2 (9.9–12.6)	6 275 (5 585–6 966)	11.0 (9.6–12.4)	0.98 (0.93–1.03)
- Sharp force (stabbing)	7 951 (6 945–8 957)	15.4 (13.3–17.6)	7 961 (7 353–8 569)	13.7 (12.4–14.9)	0.89 (0.85–0.93)
- Blunt force	4 336 (3 729–4 942)	9.0 (7.5–10.4)	3 661 (3 493–3 829)	6.5 (5.9–7.0)	0.72 (0.68–0.77)
- Strangulation/asphyxiation/suffocation	538 (463–614)	1.1 (0.7–1.4)	437 (386–488)	0.8 (0.6–1.0)	0.73 (0.61–0.87)
- Fire	203 (137–269)	0.5 (0.2–0.8)	168 (124–212)	0.3 (0.2–0.5)	0.6 (0.45–0.8)
- Poisoning/Ingestion	184 (94–275)	0.4 (0.1–0.6)	60 (46–75)	0.1 (0.1–0.2)	0.25 (0.17–0.37)
- Other^b^	240 (180–299)	0.5 (0.3–0.7)	333 (199–467)	0.6 (0.3–0.8)	1.2 (0.95–1.51)
- Unknown/Missing folder	63 (37–88)	0.1 (0.1–0.2)	581 (479–684)	1.0 (0.5–1.6)	10 (6.97–14.35)
**Transport**	**17 742 (15 366–20 118)**	**37.1 (31.1–41.2)**	**14 848 (14 375–15 321)**	**26.6 (25.2–28.1)**	**0.72 (0.7–0.74)**
All Road traffic (RT)^c^	17 103 (14 781–19 425)	36.1 (30.9–41.3)	13 939 (13 483–14 395)	25.0 (23.6–26.3)	0.69 (0.67–0.71)
- RT Pedestrian	5 604 (5 027–6 181)	11.9 (10.4–13.5)	4 563 (4 322–4 803)	8.2 (7.4–8.9)	0.69 (0.65–0.73)
- RT Passenger	4 572 (3 679–5 464)	9.3 (7.3–11.4)	3 917 (3 679–4 155)	6.9 (6.3–7.6)	0.74 (0.7–0.79)
- RT Driver	3 205 (2 655–3 755)	7.0 (5.7–8.4)	2 959 (2 783–3 135)	5.4 (4.9–5.9)	0.77 (0.72–0.83)
- RT Unspecified	3 093 (1 984–4 202)	6.3 (4.0–8.6)	1 960 (1 611–2 308)	3.5 (2.7–4.3)	0.56 (0.52–0.61)
Rail pedestrian injuries	443 (350–537)	0.9 (0.7–1.2)	307 (259–356)	0.5 (0.4–0.7)	0.56 (0.46–0.69)
Other (Transport)^d^	777 (630–925)	1.6 (1.2–2.1)	663 (619–707)	1.2 (1.0–1.4)	0.75 (0.65–0.87)
Unknown/Missing folder	48 (9–87)	0.1 (0.0–0.2)	478 (312–644)	0.9 (0.4–1.3)	9 (5.96–13.59)
**Suicide**	**6 471 (5 753–7 189)**	**13.4 (11.6–15.2)**	**6 175 (5 871–6 479)**	**11.1 (10.1–12.0)**	**0.83 (0.79–0.87)**
- Hanging	4 148 (3 613–4683)	8.4 (7.0–9.8)	4 378 (4 129–4 628)	7.7 (7.1–8.4)	0.92 (0.87–0.98)
- Poisoning (Ingestion)	1 099 (910–1 288)	2.2 (1.7–2.7)	863 (702–1024)	1.5 (1.2–1.9)	0.68 (0.6–0.77)
- Firearm	780 (653–907)	1.8 (1.3–2.2)	453 (348–557)	0.9 (0.6–1.2)	0.5 (0.43–0.59)
- Poisoning/gassing	152 (117–187)	0.3 (0.2–0.5)	76 (57–96)	0.1 (0.1–0.2)	0.33 (0.23–0.48)
- Jump from height	83 (50–117)	0.2 (0.1–0.3)	65 (41–89)	0.1 (0.1–0.2)	0.5 (0.32–0.78)
- Other^e^	198 (161–236)	0.4 (0.3–0.6)	132 (104–160)	0.3 (0.1–0.4)	0.75 (0.55–1.02)
- Unknown/Missing folder	9 (5–13)	0 (0–0)	207 (100–314)	0.4 (0.1–0.7)	-
**Other unintentional injuries**	**7 153 (6 411–7 895)**	**13.5 (11.8–15.2)**	**8 378 (7 010–9 746)**	**15.6 (12.8–18.3)**	**1.16 (1.11–1.21)**
- Fire/burns	1 973 (1 751–2 195)	4.2 (3.5–4.9)	1 734 (1 598–1 871)	3.2 (2.8–3.6)	0.76 (0.69–0.83)
- Drowning	1 690 (1 430–1 950)	3.3 (2.6–4.1)	1 541 (1 426–1 655)	2.7 (2.3–3.1)	0.82 (0.75–0.9)
- Falls	697 (572–823)	1.7 (1.2–2.1)	697 (577–817)	1.4 (1.0–1.7)	0.82 (0.71–0.95)
- Surgical and medical complications	402 (312–492)	0.9 (0.7–1.2)	1 745 (362–3 129)	3.6 (0.8–6.4)	4 (3.44–4.65)
- Poisoning (ingestion)	337 (255–419)	0.7 (0.4–0.9)	309 (264–355)	0.5 (0.4–0.7)	0.71 (0.57–0.88)
- Electrocution	267 (228–305)	0.5 (0.3–0.7)	448 (400–495)	0.8 (0.6–1.0)	1.6 (1.3–1.97)
- Lightning	258 (186–330)	0.5 (0.3–0.8)	130 (107–154)	0.2 (0.1–0.3)	0.4 (0.3–0.54)
- Suffocation/threats to breathing	205 (154–256)	0.3 (0.1–0.5)	280 (242–318)	0.5 (0.3–0.6)	1.67 (1.3–2.14)
- Other^f^	819 (676–962)	1.8 (1.3–2.2)	880 (760–999)	1.6 (1.3–1.9)	0.89 (0.78–1.02)
- Unknown/missing folder	303 (278–328)	0.7 (0.6–0.8)	613 (243–983)	1.1 (0.3–2.0)	1.57 (1.3–1.9)
**Undetermined intent**	**2 099 (1 643–2 554)**	**4.6 (3.5–5.7)**	**4 411 (3 833–4 989)**	**8.1 (6.9–9.4)**	**1.76 (1.64–1.89)**
- Poisoning/ingestion	417 (314–520)	0.9 (0.6–1.2)	631 (463–798)	1.1 (0.8–1.5)	1.22 (1.03–1.45)
- Fire/burns	283 (164–402)	0.6 (0.3–0.9)	210 (170–250)	0.4 (0.3–0.5)	0.67 (0.52–0.86)
- Other^g^	882 (711–1054)	1.8 (1.3–2.3)	1 255 (1 060–1 450)	2.3 (1.8–2.9)	1.28 (1.14–1.44)
- Unknown	517 (391–643)	1.1 (0.7–1.5)	2 316 (2 036–2 595)	4.3 (3.6–5.0)	3.91 (3.42–4.46)

*The source of the 2009 estimates is from: Matzopoulos R, Prinsloo M, Pillay-van Wyk V, Gwebushe N, Mathews S, Martin LJ, Laubscher R, Abrahams N, Msemburi W, Lombard C, Bradshaw D. Injury-related mortality in South Africa: a retrospective descriptive study of post-mortem investigations. Bulletin of the World Health Organization. 2015;93:303–13.

^a^For 2009 and 2017, the numbers for total person deaths includes 145 deaths (in 2009) and 344 deaths (in 2017) in which sex could not be determined. All estimates are weighted.

^b^For 2009, the “other” mechanisms of homicide included abandoned babies (*n* = 69), pushing (*n* = 27), drowning (*n* = 26), poisoning-gassing (*n* = 25). For 2017, the “other” mechanisms of homicide included poison-gassing (*n* = 8), concealed pregnancy/abandoned foetus (*n* = 197), maternal death/abortion-related (*n* = 5), pushed from height (*n* = 13), crushed (*n* = 13), electrocution (*n* = 5), drowning/immersion (*n* = 13) and other (*n* = 91).

^c^For 2017, “Road traffic” is a combination of Road Traffic (RT) pedestrian (*n* = 4563), RT passenger (*n* = 3917), RT Driver(*n* = 2959), RT Unspecified (*n* = 1960), Motorcycle driver (*n* = 390), Motorcycle passenger (*n* = 15) and bicycle (*n* = 134).

^d^For 2009, the “other” mechanisms of transport deaths included injuries to motorcycle riders (*n* = 377), bicycle riders (*n* = 234), railway passengers (*n* = 70), aviation (*n* = 40) and motor-cycle passengers (*n* = 19). For 2017, the “other” mechanisms of transport deaths included motorcycle riders (*n* = 390), motorcycle passengers (*n* = 15), bicycle riders (*n* = 134), railway passenger (*n* = 38), aviation casualty (*n* = 20), and other (*n* = 65).

^e^For 2009, the “other” mechanisms of suicide included sharp force injuries (*n* = 62), fire/burns (*n* = 52) and railway pedestrian injuries (*n* = 48). For 2017, the “other” mechanisms of suicide included sharp force injuries (*n* = 64), fire/burns (*n* = 35), railway pedestrian injuries (*n* = 14) and other (*n* = 19).

^f^For 2009, the “other” mechanisms of unintentional injury includes blunt-force injuries (*n* = 29), crushing (*n* = 186), poisoning/gassing (*n* =  130 deaths), environmental exposure (*n* =  121), animal contact (*n* = 111 deaths), circumcision (*n* = 83), sharp force injuries (*n* = 48 deaths), gunshot injuries (*n* = 48), injuries from machinery (*n* = 26) and explosive blasts (*n* = 19). For 2017, the “other” mechanisms of unintentional injury includes firearm discharge (*n* = 17), sharp force (*n* = 42), blunt force (*n* = 218), poison (gassing) (*n* = 75), crushing (*n* = 174), animal contact (*n* = 104), machinery (farm/recreational) (*n* = 29), natural/environmental (*n* = 142), explosive blast (*n* = 10), circumcision (*n* = 34), SIDS (*n* = 18), exposure/hypothermia (*n* = 40), maternal death/abortion-related (*n* = 80), mining (*n* = 113) and other (*n* = 48).

^g^For 2009, “other” mechanisms of undetermined manner included blunt force (*n* = 273), drowning (*n* = 125), gunshot injuries (*n* =  87), poisoning/gassing (*n* = 85), falls (*n* = 69), hanging (*n* = 66), strangled (*n* = 37), surgical and medical complications (*n* = 23), sharp force injuries (*n* = 22), electrocutions (*n* = 21) and transport deaths of undetermined manner (*n* = 10). For 2017, “other” mechanisms of undetermined intent included firearm discharge (*n* = 24), abandoned baby (*n* = 51), sharp force (*n* = 20), MV pedestrian (*n* = 4), blunt force (*n* = 328), asphyxiated/strangled (*n* = 76), hanging (*n* = 124), poison/gassing (*n* = 46), explosive blast (*n* = 2), fall/push/jump (*n* = 64), drowning/immersion (*n* = 88), railway passenger (*n* = 14), maternal death/abortion-related (*n* = 12), other (*n* = 402).

Homicide and transport deaths, specifically road traffic mortality, continued to be the leading manner for injury deaths in 2017. Nearly half (48.1%) of all injury deaths were intentional (i.e. homicide and suicide), of which homicide accounted for more than a third (19 477 deaths). The homicide rate decreased significantly, from 38.4 per 100 000 in 2009 to 34 per 100 000 population in 2017 (IRR = 0.89). While there was no change in the firearm homicide rate, there was an increase in firearms as a proportion, accounting for nearly a third of all mechanisms for homicide. Apart from firearms, there was a significant reduction in the specified homicide mechanisms (IRR estimates in Table 1).

As the second leading manner of death, the 13 939 road traffic deaths accounted for 26.2% of all injury deaths in 2017. The road traffic mortality rate for 2017 decreased significantly between 2009 and 2017, from 36.1 to 25.0 per 100 000 population (Table 1), effectively a rate decrease of 31% (IRR = 0.69). Pedestrian deaths accounted for approximately one-third, followed by passenger (28.1%) and driver deaths (21.2%). A significant decrease in the mortality rates was noted for all three aforementioned categories (see IRRs in Table 1).

There was a significant increase in unintentional injuries, from 13.5 to 15.6 per 100 000 (IRR = 1.16), and a significant decrease for suicide, from 13.4 to 11.1 per 100 000 (IRR = 0.83). A further subgroup of deaths was classified as ‘undetermined intent’ (4 411 or 8.3% of injury deaths in 2017).

In 2009, homicide rates were highest among the 30–44-year age group (Table 2). By 2017, the peak had shifted to the younger 15–29 age group, with a rate of 57.0 per 100 000, with no significant change for this age group (IRR = 1.01). The homicide rate for children 5–14 years decreased significantly by 40% (IRR = 0.6) and was lower (1.9 per 100 000 in 2017) than for children 0–4 years (4.2 per 100 000).

**Table 2.** Homicide, suicide, road traffic, and other unintentional injury mortality for 2009 and 2017 by age group (weighted).

Manner of death by age group	2009		2017		
	N (95% CI)	Mortality rate per 100,000 population (95%CI)	N (95% CI)	Mortality rate per 100,000 population (95%CI)	Incidence rate ratio: 2017/2009: (95% CI)
**Homicide (X85-Y09)**	**19028 (16852–21204)**	**38.4 (33.8–43.0)**	**19477 (18153–20800)**	**34.0 (31.7–36.3)**	**0.91 (0.88–0.93)**
0–4	286 (243–328)	5.2 (4.4–6.0)	242 (158–326)	4.2 (2.8–5.7)	0.81 (0.64–1.03)
5–14	287 (172–401)	3.1 (1.8–4.3)	193 (166–219)	1.9 (1.6–2.1)	0.6 (0.47–0.78)
15–29	8531 (7466–9596)	56.7 (49.6–63.8)	8523 (8027–9019)	57.0 (53.7–60.3)	1.01 (0.96–1.05)
30–44	5855 (5346–6365)	58.7 (53.6–63.8)	6777 (6283–7272)	52.5 (48.7–56.3)	0.89 (0.85–0.94)
45–59	2295 (2043–2547)	35.9 (32.0–39.9)	2283 (2150–2416)	30.4 (28.6–32.2)	0.85 (0.78–0.92)
60+	995 (822–1169)	25.2 (20.8–29.6)	969 (898–1041)	19.6 (18.1–21.0)	0.78 (0.69–0.88)
**Road-traffic (V00-V89)**	**17103 (14781–19425)**	**36.1 (30.9–41.3)**	**13 939 (13483–14395)**	**25.0 (23.7–26.3)**	**0.72 (0.7–0.75)**
0–4	741 (616–867)	13.5 (11.2–15.8)	399 (364–433)	7.0 (6.3–7.5)	0.51 (0.43–0.61)
5–14	1048 (878–1218)	11.2 (9.4–13.1)	730 (666–794)	7.0 (6.4–7.6)	0.62 (0.55–0.71)
15–29	5454 (4696–6211)	36.3 (31.2–41.3)	4308 (4133–4483)	28.8 (27.6–30.0)	0.79 (0.75–0.84)
30–44	5179 (4459–5899)	52.0 (44.7–59.2)	4836 (4613–5058)	37.5 (35.7–39.2)	0.72 (0.68–0.76)
45–59	2762 (2396–3128)	43.2 (37.5–49.0)	2357 (2246–2468)	31.4 (29.9–32.9)	0.73 (0.67–0.78)
60+	1270 (1089–1452)	32.2 (27.6–36.8)	1151 (1069–1234)	23.3 (21.6–24.9)	0.72 (0.65–0.81)
**Suicide (X60-X84)**	**6471 (5753–7189)**	**13.4 (11.6–15.2)**	**6175 (5871–6479)**	**11.1 (10.2–11.9)**	**0.85 (0.81–0.89)**
5–14	127 (94–160)	1.4 (1.0–1.7)	180 (149–211)	1.7 (1.4–2.0)	1.27 (0.93–1.74)
15–29	2652 (2332–2971)	17.6 (15.5–19.8)	2306 (2158–2453)	15.4 (14.4–16.4)	0.87 (0.81–0.95)
30–44	2159 (1919–2399)	21.7 (19.3–24.1)	2123 (1996–2251)	16.4 (15.5–17.4)	0.76 (0.7–0.83)
45–59	1056 (910–1203)	16.5 (14.2–18.8)	985 (889–1080)	13.1 (11.8–14.4)	0.79 (0.7–0.9)
60+	361 (295–428)	9.2 (7.5–10.8)	528 (469–588)	10.7 (9.5–11.9)	1.17 (0.97–1.41)
**Other unintentional injuries (W00-X59)**	**7153 (6411–7895)**	**13.5 (11.8–15.2)**	**8378 (7010–9746)**	**15.5 (12.8–18.3)**	**1.04 (1–1.09)**
0–4	1045 (895–1194)	19.0 (16.3–21.8)	954 (851–1057)	16.6 (14.8–18.4)	0.87 (0.77–0.99)
5–14	708 (603–814)	7.6 (6.5–8.7)	690 (635–746)	6.6 (6.1–7.2)	0.87 (0.75–1.01)
15–29	1702 (1513–1891)	11.3 (10.1–12.6)	1703 (1458–1947)	11.4 (9.8–13.0)	1.01 (0.92–1.11)
30–44	1534 (1408–1659)	15.4 (14.1–16.6)	2071 (1786–2355)	16.0 (13.8–18.2)	1.04 (0.95–1.14)
45–59	1035 (924–1146)	16.2 (14.5–17.9)	1311 (1083–1539)	17.5 (14.4–20.5)	1.08 (0.96–1.21)
60+	856 (746–966)	21.7 (18.9–24.5)	1523 (1077–1970)	30.8 (21.8–39.8)	1.42 (1.26–1.59)

A significant decrease in age-standardised road traffic mortality rates, as shown in Table 1, was evident across all age groups (Table 2). A significant 28% decrease in the 30–44-year age group’s road traffic mortality rate was observed between 2009 and 2017 (IRR = 0.0.72), but the rate (37.5 per 100 000) remained highest of all age groups. The 0–4 and 5–14-year age groups had the largest percentage decrease (49.0% and 38.0% respectively).

For suicide, the rates remained highest in the 15–29 and 30–44-year age groups in 2017. The peak was among the 30–44-year age group, despite a significant 24.0% decrease (IRR = 0.76) in the rate, from 21.7 to 16.4 per 100 000 population. A significant increase in the other unintentional injury mortality rate was noted for the 60+ age group (IRR = 1.42). Among children under-15 years, the other unintentional injury mortality rate remains highest for the 0–4-year age group (16.6 per 100 000).

When comparing our 2017 survey data with the GBD estimates for South Africa (Table 3), we found notable similarities at the higher-level all-injury comparison. The 2017 GBD injury mortality rate of 97.4 per 100 000 was similar to our survey’s 95.3 per 100 000 population. However, our survey’s adjusted homicide rate comparison with that of the GBD was higher. A notable difference was observed for firearm homicide, where our survey’s rate of 12.0 per 100 000 was more than double the GBD firearm homicide rate of 5.2 for South Africa. On the contrary, our survey reported lower rates for suicide and road traffic mortality, compared to the GBD estimates for South Africa. Overall, our survey indicated that South Africa had much higher rates of homicide and firearm homicide compared to the global average estimated by the GBD.

**Table 3.** 2017 adjusted injury mortality survey and GBD injury mortality rates (per 100 000 population).

	2017 Rate (95% CI)	2017 Adjusted^h^ Rate (95% CI)	2017 GBD^i^ Rate for South Africa (95% CI)	2017 Adjusted^h^: GBD SA Ratio	2017 GBD Global^i^ Rate (95% CI)	2017 IMS Adjusted^h^: GBD Global Ratio
**All injuries**	95.3 (89.7–100.9)	95.3 (89.7–100.9)	97.4 (86.4–109.0)	1.07	55.6 (50.7–59.6)	1.87
**Homicide**	34.0 (31.6–36.4)	37.1 (34.2–40.1)	33.3 (28.5–38.5)	1.11	5.5 (5.2–5.9)	6.75
- Firearm	11.0 (9.6–12.4)	12.0 (10.4–13.6)	5.2 (4.3–6.8)	2.31	2.4 (2.3–2.6)	5.00
**Suicide**	11.1 (10.1–12.0)	12.1 (11.0–13.2)	13.4 (10.9–16.4)	0.90	9.8 (9.0–10.6)	1.23
**Transport**	26.6 (25.2–28.1)	29.1 (27.3–30.9)	33.4 (27.1–37.8)	0.87	16.8 (14.7–18.1)	1.73
- Road traffic	25.0 (23.6–26.3)	27.3 (25.6–29.0)	32.4 (26.3–36.6)	0.84	15.7 (13.8–16.9)	1.74
**Other unintentional**	15.6 (12.8–18.3)	17.0 (13.9–20.2)	17.0 (15.2–18.8)	1.00	23.0 (20.2–25.0)	0.74
**Undetermined**	8.1 (6.9–9.4)	–	–	–	–	–

^h^2017 Survey adjusted rate represents the rates when the undetermined manner of death was proportionally redistributed.

^i^The GBD data for South Africa and the Global estimates were downloaded from: Global Burden of Disease Collaborative Network. Global Burden of Disease Study 2017 (GBD 2017) Results. Seattle, United States: Institute for Health Metrics and Evaluation (IHME), 2020. Available from https://vizhub.healthdata.org/gbd-results/.

## Discussion

The comparison of the South African injury mortality rates for 2009 and 2017 indicates some progress towards achieving the SDGs. The significant decrease in the overall injury mortality rate from 109 to 95 per 100 000 between 2009 and 2017 appears to largely be driven by the significant 31% decrease in road traffic mortality rates. The significant reduction in road traffic mortality across all age groups indicates that South Africa is well on its way towards meeting the SDG Target 3.6 for road safety [6] and have been validated against national findings from the Road Traffic Management Corporation [24,32]. Contrary to the positive outlook for road safety targets, the SDGs targeting the reduction of violence and related death rates; suicide as an indicator for pre-mature mortality; and to end newborn and under-5 mortality needs more intense preventative approaches.

The 11.0% decrease in the national homicide rate, although significant, had seen virtually no change in firearm homicide, a leading contributory mechanism, as we track progress to meet SDG Target 16.1 to reduce violence. An analysis of South African Police Service (SAPS) crime statistics indicates that a recent increase in the national homicide rate, to 45 per 100 000 population, was driven by the four largest populated provinces: the Eastern- and Western Cape, Gauteng, and KwaZulu-Natal [33]. With homicide as the major contributor to injury mortality in South Africa, an analysis of sex-disaggregated homicide risk using data from this study identified the disproportionate burden by adult males and called for a redoubling of efforts to control alcohol and firearms [18]. The consistently high firearm homicide rate highlights the need for firearms legislation reforms and intervention strategies that target the risk factors for violence, particularly among the 15–29-year age group. A 2009 analysis of firearm homicide risk, adjusting for age, sex, and race differences identified a 2.6 times significantly higher risk in urban compared to rural areas [34], which identifies some target areas for intervention. Enforcement of the Firearms Control Act was associated with a reduction in homicide rates between 2004 and 2010, but poorly sustained efforts resulted in an increase in rates for subsequent years [35]. In South Africa, alcohol has largely been ascribed as a risk factor to male homicide [36], and restrictions on alcohol sales during the COVID-19 pandemic demonstrated its effectiveness to reduce the trauma caseload presenting to hospitals [37].

Progress towards meeting SDG Target 3.4, to reduce premature mortality from non-communicable diseases, is indicated by the 17.0%, significant decrease in the suicide rate. The increase in the suicide rate for children under-15 years is concerning. A causal relationship is well established between all forms of child maltreatment, intimate partner violence (IPV), bullying victimisation, and mental health disorders [38–40]. A South African risk factor study for 2012 identified that 53% of the depressive disorders burden in males and 65% in females was attributable to interpersonal violence, which included the joint effect of exposure to child maltreatment, bullying, and other community violence in males, but in females included the additional contribution of IPV and sexual violence by non-partners [41]. Improved identification for those at risk should be encouraged [39] to protect children against violence and should include those younger than 15 years.

The assessment of progress towards SDG Target 3.2, to end preventable deaths of newborns and children under-5 years, showed that the highest rates of under-5 injury mortality remain in the other unintentional injury category, which are 2.5 times higher than for children 5–14 years. For the under-5’s, road traffic deaths had the largest decrease, but the greater vulnerability to homicide for children 0–4 years persist since the early 2000’s [42], in comparison to children 5–14 years. Promising interventions to prevent burns and drowning as the leading mechanisms for other unintentional injuries, and childhood road traffic safety strategies should be identified and refined for sustained effectiveness. An in-depth investigation of child homicides in South Africa in 2009 found that the majority of deaths were under the age of 5 years, including neonates abandoned at birth, and that the main perpetrators were parents and family members [21]. An updated analysis comparing child abuse-related murders between 2009 and 2017 showed a significant decrease in child abuse murders and although children under 5 years remained the most vulnerable, the largest decrease was among this youngest group. Parents remained the main perpetrators with an increase in fathers as perpetrators found in 2017 compared to 2009 [22].

The global target for road safety, which could not be reached by the set deadline, was renewed for a second Decade of Action for 2021–2030 [43]. There is a need for the investigation and identification of road safety interventions that may have been in place before the South African government adopted the global plan for road safety [44] in its 2016–2030 national road safety strategy. This plan utilises the evidence-based principles of the global ‘Safe Systems Approach’, which aims to guide the building of roads in a manner that prevents crashes, and in the event, to ensure that the force of impact does not result in severe injury or death. It also aims to ensure that those who are injured are rescued and receive adequate trauma care [45–47]. It is a concept that implies shared responsibility between governments, private sector, civil society, engineers, health professionals, road users, and others, to enable a safer transport system. This is to ensure that interventions go beyond behaviour change via education and enforcement, and instead address underlying factors such as mobility-planning to promote safe, environment-friendly travel, setting and management of safe speeds, intersection design for safe pedestrian crossing, road design that accounts for human error, improved public transport, safe vehicle design, and improved coordination and quality of post-crash emergency response and care [48]. What may have contributed to the significant decrease in road traffic deaths is not yet known, but as the main risks to road traffic deaths are speed and alcohol, the deterioration of South Africa’s roads [49,50] may have contributed to slower speeds in anticipation of potholes and non-tarred roads in rural areas. In recent years, road traffic law enforcement was intensified to curb driving under the influence and reckless driving, which led to arrests across provinces. In addition, road safety awareness campaigns were implemented in schools and the post-crash response is being addressed. However, by 2022 only 57% of the planned, short-, medium- and long-term national road safety strategies were fully implemented, with the remainder either undergoing development or had not yet reached the planning stage [51].

The fact that the lowest decline in road traffic mortality was for the 15–29-year age group indicates the need for interventions to be especially focused on both young and novice drivers, along with road safety strategies for young and vulnerable road users. In tandem with the safe systems approach to road safety, these interventions should be based on global, evidenced-based strategies focused on the *S*ave *LIVES* package: *S*peed management (i.e. through legislation and road modification); *L*eadership on road safety; *I*nfrastructure design and improvement (i.e. sidewalks, bicycle lanes, safe crossings); *V*ehicle safety (i.e. standard regulations for seat belts, anchorages, and child restraint points); *E*nforcement of traffic laws (i.e. drinking and driving, securing of children in child restraint seats, motorcycle helmets); and *S*urvival (i.e. integrated emergency care systems and emergency responder training) [52]. Should the government successfully implement these evidence-based strategies to road safety, it is expected that road traffic mortality rates will continue to decrease beyond this survey, and if not, current intervention strategies should be evaluated and adjusted.

Our study highlights the importance of empirical data that can identify discrepancies in widely available and highly utilised global estimates, which are often relied upon to prioritise injury prevention strategies in LMICs with no reliable injury surveillance systems in place. After adjusting for deaths classified as undetermined manner in our survey, the GBD estimates for homicide in SA remained 10% lower, while their estimates for suicide and road traffic mortality rates were 10% and 16% higher, respectively, than our 2017 findings.

Our study has some limitations. Firstly, the different sampling strategies used in the two survey years (metro/non-metro vs provincial sampling) could not enable direct provincial comparisons. However, it is important to note that both surveys were nationally representative. In addition, we utilise data for two time points and cannot make any inference to trends. Nevertheless, our survey’s cause profile is more defined, to that reported through official national vital registration data, where nearly 70% of injury-related deaths are reported as ‘other unintentional injuries’. Our estimates have been reliably validated [24] against alternative data sources for homicide and road traffic mortality from the SA Police Service and the Road Traffic Management Corporation, as well as a national study on cause of death validation through clinicians’ review and ICD-10 coding from post-mortem records [53]. In the absence of reliable vital registration data for injury deaths, our study identifies the leading manner of death and injury mechanisms, to inform injury prevention policy and practice.

## Conclusion

This study provides valuable insights into South Africa’s progress towards meeting key Sustainable Development Goals related to injury mortality. While commendable strides have been made in reducing road traffic mortality rates, particularly among pedestrians, challenges persist in achieving targets related to young and novice drivers, and to identify which interventions were successful. For violence reduction, interventions should be intensified for the 15–29-year age group. The findings underscore the importance of sustained efforts in implementing evidence-based strategies, such as the Safe Systems Approach to road safety, firearms legislation reforms, and other interventions targeting risk factors for violence. To effectively meet SDG targets, there is a pressing need for comprehensive evaluations of programmes such as the 2016–2030 national road safety strategy, to identify successful interventions and areas for adjustment. The study findings call for integrated approaches to protect children from violence and promote their mental well-being. Our study has shown the value of surveillance in the absence of reliable routine data, which also ensure we are not reliant on modelled GBD estimates. Survey data allow us to accurately assess the country’s progress towards the SDGs through commitment to evidence-based policymaking.
